# Steroid Receptor Isoform Expression in *Drosophila* Nociceptor Neurons Is Required for Normal Dendritic Arbor and Sensitivity

**DOI:** 10.1371/journal.pone.0140785

**Published:** 2015-10-23

**Authors:** Aidan L. McParland, Taylor L. Follansbee, Gwendolyn D. Vesenka, Alexandra E. Panaitiu, Geoffrey K. Ganter

**Affiliations:** Department of Biology, College of Arts and Sciences, University of New England, Biddeford, Maine, United States of America; CNRS UMR7622 & University Paris 6 Pierre-et-Marie-Curie, FRANCE

## Abstract

Steroid hormones organize many aspects of development, including that of the nervous system. Steroids also play neuromodulatory and other activational roles, including regulation of sensitivity to painful stimuli in mammals. In *Drosophila*, ecdysteroids are the only steroid hormones, and therefore the fly represents a simplified model system in which to explore mechanisms of steroid neuromodulation of nociception. In this report, we present evidence that ecdysteroids, acting through two isoforms of their nuclear ecdysone receptor (EcR), modulate sensitivity to noxious thermal and mechanical stimuli in the fly larva. We show that EcRA and EcRB1 are expressed by third instar larvae in the primary nociceptor neurons, known as the class IV multidendritic neurons. Suppression of EcRA by RNA interference in these cells leads to hyposensitivity to noxious stimulation. Suppression of EcRB1 leads to reduction of dendritic branching and length of nociceptor neurons. We show that specific isoforms of the ecdysone receptor play critical cell autonomous roles in modulating the sensitivity of nociceptor neurons and may indicate human orthologs that represent targets for novel analgesic drugs.

## Introduction

Seeking treatment for pain is one of the top reasons patients visit their physicians [1]. For many years researchers have investigated the mechanisms of ailments that may be causing pain, but have in some ways neglected to consider pain as a treatable malady itself. The incidence of idiopathic acute and chronic pain is almost as prevalent as pain associated with a different causative ailment, suggesting that pain should be viewed not only as a symptom, but also as a condition on its own.

The fruit fly represents a simplified model in which to study pain, and one with important similarities to mammalian systems. The *Drosophila* model is free of placebo-induced analgesia [2,3], making it ideal for the investigation of baseline nociceptive behavior. Other nervous abnormalities including Parkinson’s disease, Huntington’s disease and Alzheimer’s disease have all been studied using the fruit fly model [4].

Multiple studies have investigated the role of steroids in modulating the sensation of pain and avoidance response in vertebrates [5–8]. However, because there are dozens of steroid hormones in vertebrate systems, some of which may play stimulatory or antagonistic roles with each other, it has been difficult to investigate the combined effect of multiple hormones on pain response or sensation. Future studies on the endocrine regulation of pain would benefit from a system that is simplified and genetically tractable. In *Drosophila*, ecdysteroids are the only steroid hormones and are responsible for regulating a diverse array of behaviors, including courtship, memory, and, as shown in this study, responses to noxious stimuli.

Ecdysone binds to the nuclear ecdysone receptor (EcR), a transcription factor consisting of three isoforms: EcRA, EcRB1 and EcRB2. Each isoform genomically regulates a diverse array of processes. For example, EcRB1 appears to be involved in controlling motor neuronal growth in a cell specific manner, whereas EcRB2 is responsible for the elaboration of the central dendritic arbor [9]. Steroid signaling in *Drosophila* regulates transitions between developmental stages and, via specific EcR isoforms, is responsible for the remodeling of neurons during metamorphosis. Steroid signaling has also been shown to perform a similar role in vertebrates, for example during puberty in mammals [10,11], when steroids regulate the innervation of motor neurons and the growth of sexually dimorphic muscles [12].

Previous studies have implicated EcR in the modulation of dendritic architecture of sensory neurons in *Drosophila* larvae. EcR is known to be required for normal growth of the non-nociceptive larval class I sensory neurons. When EcR expression is impaired in these cells not thought to play a role in nociception, a reduced dendritic arbor results [13]. EcR signaling is known to regulate dendritic and axonal pruning of larval neurons during metamorphosis [14]. A heterodimer composed of isoform EcRB1 and EcR co-receptor ultraspiracle (USP) is necessary to regulate the pruning process in which all dendrites are removed in preparation for new outgrowth to innervate the adult integument [14]. In the central nervous system, the gamma neurons of the mushroom body, deprived of EcRB1, fail to prune, and ectopic expression of EcRB1 does not induce pruning, suggesting that while EcRB1 is necessary for pruning of the gamma neurons, other pathways are also necessary for this process [15]. Although not much is known about the pruning machinery downstream of EcRB1, Kirilly and coworkers have identified the transcription factor Sox14 to be essential for both axonal and dendritic pruning [16].

Despite these indications of ecdysteroid importance in sensory neurons, little is currently known about the roles each EcR isoform may play in the development of the nociceptor cells’ dendritic arbors and in modulating the fly’s response to noxious stimuli. We have primarily focused this investigation on the cell-autonomous roles of EcRA, EcRB1 and USP in dendritic morphology and in regulating avoidance behavior in response to noxious stimuli. We impaired the ecdysone signaling system by cell-specific RNAi in the nociceptive class IV multidendritic neurons of *Drosophila* third instar larvae. The dendritic arbors of these neurons were analyzed, as were the behavioral responses of these animals to noxious thermal and mechanical stimuli. The results indicate that the EcRB1 and EcRA isoforms of the nuclear ecdysone receptor are required, in an otherwise wild-type animal, for normal dendritic morphology and for maximal behavioral response to noxious stimulation, respectively.

## Results

**Fig 1:**
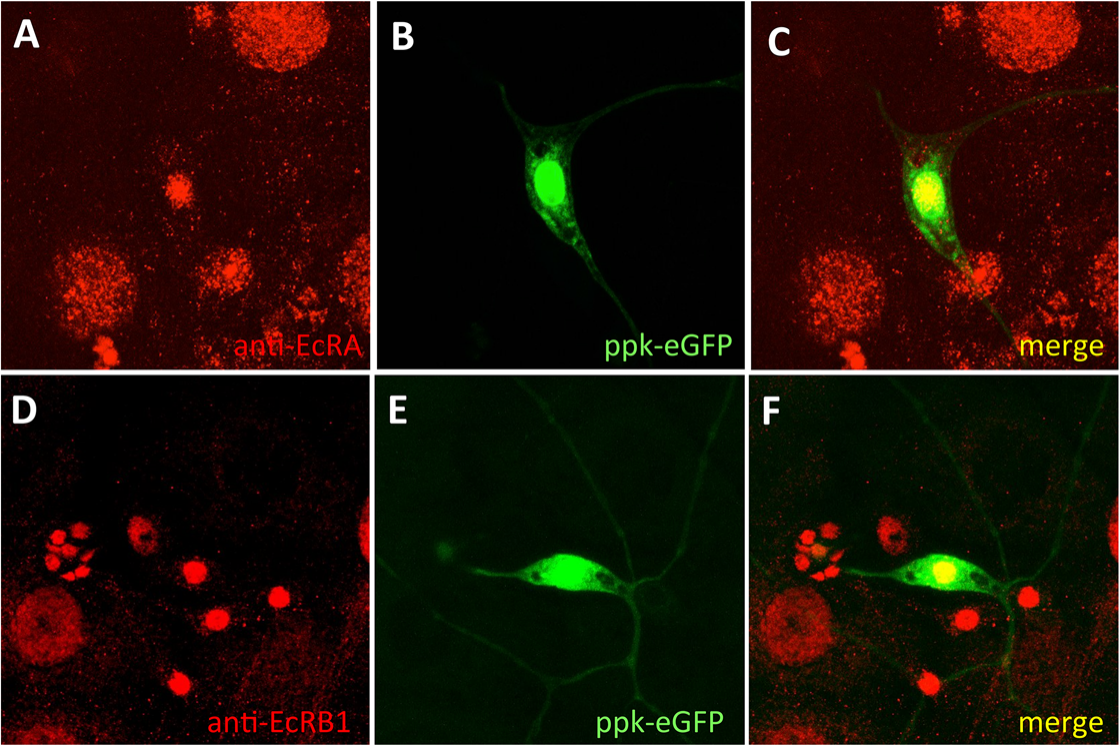
EcRA and EcRB1 are expressed in the class IV primary nociceptor neurons. (A) *ppk-eGFP* larva stained with anti-EcRA. (B) The class IV neuron is identified by eGFP expression in *ppk-eGFP*. (C) Merge of (A and B) indicates the presence of EcRA in the nociceptor neurons. (D) Sensory cluster of *ppk-eGFP* larva stained with anti-EcRB1. (E) Nociceptor neuron of same cluster is identified by eGFP expression in *ppk-eGFP*. (F) Merge of (D and E) indicates the presence of EcRB1 in the class IV nociceptor neurons.

Both EcRA and EcRB1 isoforms can be detected in the class IV multidendritic neurons (Fig 1) via immunohistochemistry. The identity of these nociceptor neurons was indicated by their fluorescence, achieved using *ppk-eGFP*, a fusion of the green fluorescent protein (eGFP) reporter to the promoter of the DEG-ENaC channel *pickpocket* (*ppk*) [17]. Using isoform-specific monoclonal antibodies, we show that in third instar larvae, EcRA and EcRB1 expression is most obvious in the nuclei of these cells (Fig 1).

**Fig 2:**
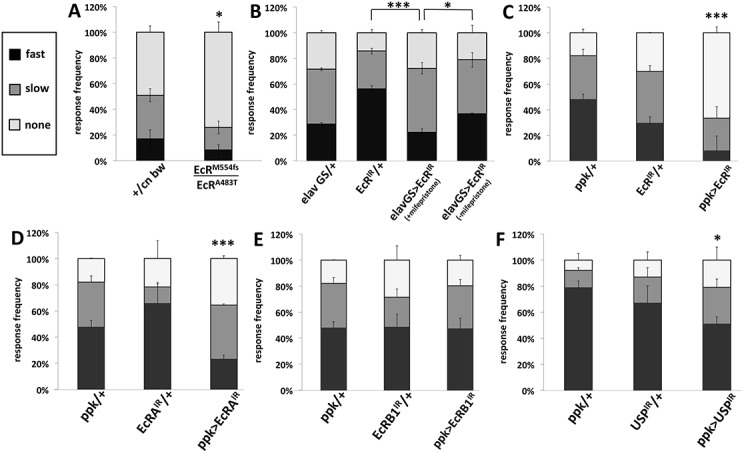
Ecdysone receptor mutants are less sensitive to noxious thermal stimulation. Foraging third instar larvae were gently touched on their dorsal surface with a probe at 45°C. Larvae responding with a nocifensive roll were classified as fast (<6 seconds) or slow (between 6 and 20 seconds), or nonresponders if they did not respond within 20 seconds. Distributions were compared using Fisher’s exact test. Asterisks indicate statistically different results (* is p<0.05, *** is p<0.001). N > 90. (A) Mutants bearing one null allele (*EcR* ^*M554fs*^) and one temperature sensitive allele (*EcR* ^*A483T*^) were significantly less sensitive than controls (+ = Canton-S) comprising the genetic backgrounds of each mutation. (B) *EcR* RNAi was driven by the mifepristone-inducible neuron-specific *elav-Geneswitch*. (C-F) Various *EcR* and a *USP* RNAi were driven by *ppk1*.*9-Gal4*, specific to the nociceptive class IV multidendritic neurons.

**Fig 3:**
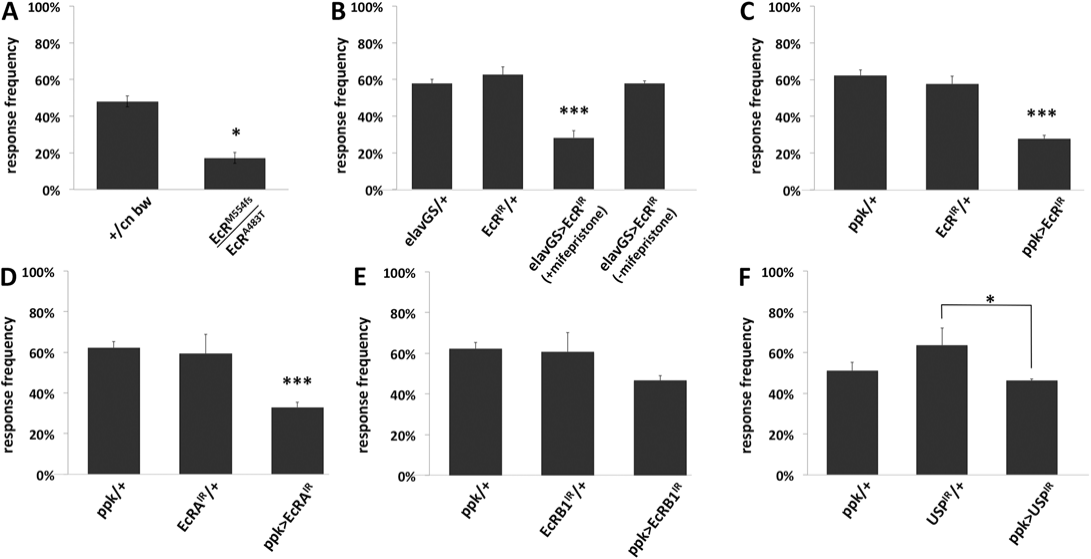
Ecdysone receptor mutants are less sensitive to noxious mechanical stimulation. Foraging third instar larvae were stimulated on their dorsal surface with a von Frey filament calibrated to deliver 45 mN. Larvae responding with a nocifensive roll were classified as responders. Distributions were compared with Fisher’s exact test. Asterisks indicate statistically different groups (* is p<0.05, *** is p<0.001). N > 90. (A) Mutants bearing one null allele of EcR (EcR^M554fs^) and one temperature sensitive allele (EcR^A483T^) were significantly less sensitive than controls comprising the genetic backgrounds of each mutation (+ = Canton-S). (B) EcR RNAi is driven by the mifepristone-inducible neuron-specific elav-Geneswitch. (C-F) Various EcR and a USP RNAi are driven by ppk1.9-Gal4, specific to the nociceptive class IV multidendritic neurons.

The activity of EcR was conditionally reduced throughout the third-instar larva using the temperature-sensitive allele, *EcR* ^*A483T*^ [18]. The behavioral responses to noxious stimulation were measured following 24 hours at the restrictive temperature (29°C). Compared to controls, larval *EcR* mutants were hyposensitive to both thermal (Fig 2A) and mechanical (Fig 3A) stimulation. The morphology of class IV multidendritic neurons in these mutants was not investigated.

EcR expression was suppressed using RNAi under the control of the neuron-specific promoter of *elav* driving the progesterone-inducible GAL4 Geneswitch [19]. When *EcR* RNAi suppression was induced by the progesterone analog mifepristone for 24 hours, third-instar larvae became hyposensitive to noxious mechanical stimulation (Fig 3B), but the hyposensivity observed upon noxious thermal (Fig 2B) stimulation was not significantly different from all controls. The morphology of class IV multidendritic neurons in these genotypes was not investigated.

**Fig 4:**
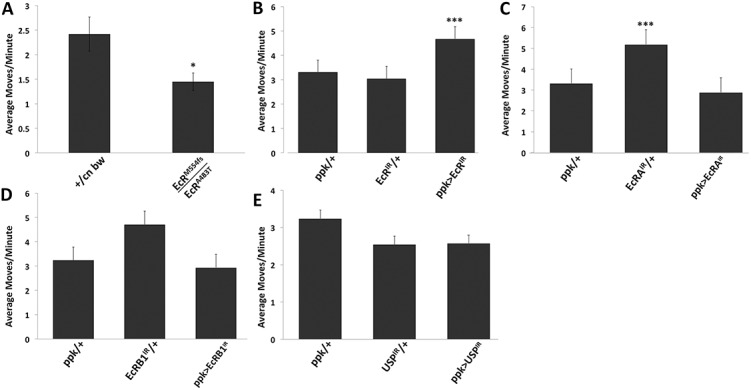
Analysis of ecdysone receptor mutant locomotion. Third instar larvae were assayed for spontaneous locomotor activity using the *Drosophila* activity monitor (DAM). Data were collected as moves per minute for each larva and averaged over a twenty-minute period. A Students t-test was used to determine statistical significance (** indicates p<0.01, *** indicates p<0.001), n = 32 per genotype. (A) Mutants bearing one null allele of *EcR* (*EcR* ^*M554fs*^) and one temperature sensitive allele (*EcR* ^*A483T*^) were significantly less motile (p<0.011) than controls comprising the genetic backgrounds of each mutation (+ = Canton-S). (B) Locomotor activity was increased in larvae in which all EcR isoforms were suppressed specifically in nociceptor neurons. (C-E) The spontaneous locomotion of various other EcR and a USP RNAi driven by *ppk-Gal4* were not significantly different from both controls.

Spontaneous locomotor activity was measured using the *Drosophila* activity monitor (DAM, [20]) (Fig 4). Larvae with EcR reduced globally following 24 hours at the restrictive temperature for *EcR* ^*A483T*^, showed reduced locomotion compared to controls (Fig 4A). When total EcR or individual isoforms were silenced in nociceptor neurons via RNAi (Fig 4B–4E), the activity of the EcR mutant, but not the other genotypes, was significantly different from both controls.

**Fig 5:**
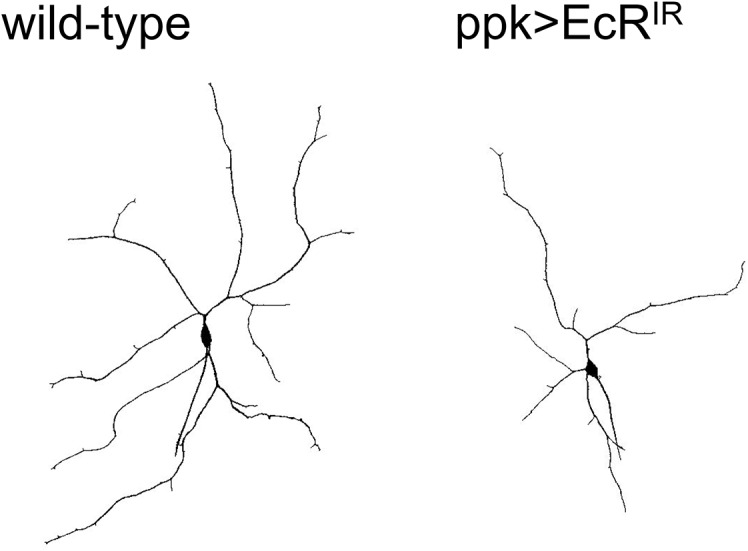
Nociceptor neuron dendritic reduction in EcR RNAi larvae. Confocal visualization of live third instar larvae expressing eGFP in nociceptor neurons (*ppk-eGFP*; left) reveals reduction in dendritic length and branch number when EcR is suppressed via RNAi driven by ppkGal4 (right). The ddaC neurons depicted represent the average for overall dendritic length, and more than 70% of all neurons analyzed (n = 20 for wild-type and n = 19 for EcR-RNAi) showed a length that was within one standard deviation from the lengths of these representative neurons.

**Fig 6:**
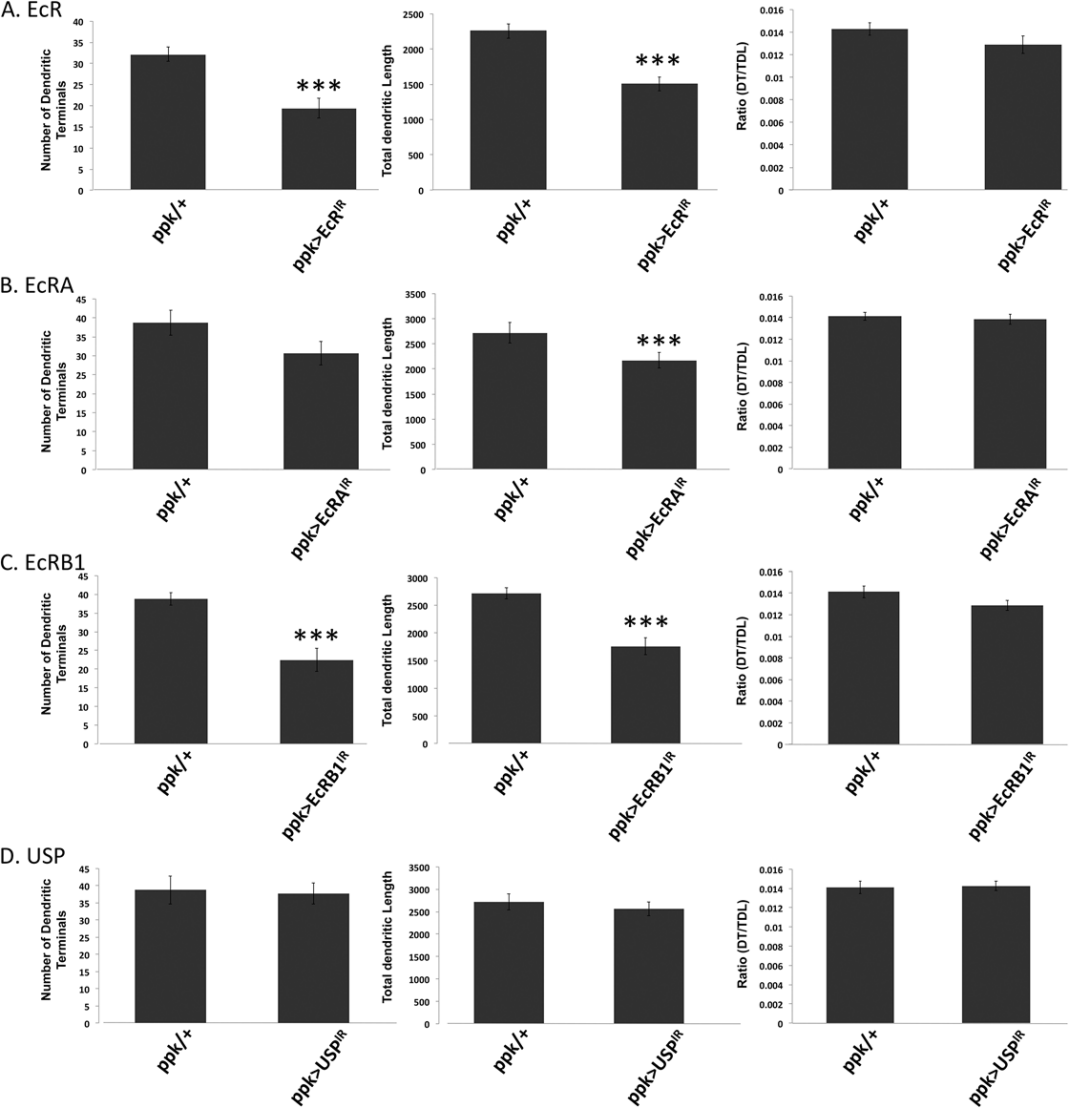
Morphometric analysis of class IV neurons in EcR, EcRA, EcRB1 and USP mutant flies. All neurons in the analysis were class IV ddaC neurons from third instar larvae. Experimental animals were compared to control animals for number of dendritic terminals (left), total dendritic length (center) and the ratio of dendritic terminals/total dendritic length as a measure of complexity (right). (A) *EcR* mutants have significantly fewer dendritic terminals and a reduced dendritic length, but maintain the same complexity. Control n = 20, *EcR* mutant n = 19. (B) *EcRA* mutants show reduced dendritic length, but both the number of dendritic terminals and complexity remain the same. Control n = 18, *EcRA* mutant n = 19. (C) *EcRB1* mutants show a reduced number of dendritic terminals and reduced dendritic length, but are similar in complexity. Control n = 18, *EcRB1* mutant n = 17. (D) *USP* mutant animals show no difference in number of dendritic terminals, total dendritic length or complexity. Control n = 18 *USP* mutant n = 16. *** indicates p < 0.001.

Expression of all isoforms of EcR was suppressed via cell-specific RNAi in the class IV multidendritic neurons, which are known to detect noxious thermal and mechanical stimuli [21]. Significant reduction in sensitivity of larvae to noxious thermal (Fig 2C) and mechanical (Fig 3C) stimuli was observed. Dendrite branch number and overall dendritic length in the most dorsal class IV cells, ddaC, were found to be significantly reduced in larvae with reduced EcR (Figs 5 and 6A). The ratio of branch number to length was not significantly altered, meaning that EcR hypomorphy produces a dendritic arbor with proportions similar to controls but reduced in scale.

We used RNAi to specifically suppress the expression of EcRA and EcRB1 isoforms in the nociceptor neurons. EcRB2 was not analyzed because RNAi tools targeting this isoform are currently unavailable. When the expression of the EcRA isoform was reduced in these neurons, larvae were rendered significantly hyposensitive to noxious thermal (Fig 2D) and mechanical (Fig 3D) stimuli. Morphometric analysis of the dendritic field of ddaC neurons revealed a significant reduction in dendritic length but not branch number (Fig 6B).

When the expression of the EcRB1 isoform was suppressed in the class IV neurons, larvae showed no significant changes in sensitivity to noxious thermal (Fig 2E) or mechanical stimuli (Fig 3E). On the other hand, the dendritic fields of EcRB1-deficient class IV ddaC neurons featured a significant reduction in both branch number and overall length (Fig 6C). As was the case in EcR RNAi larvae (Fig 6A right), the ratio of branch number to length was not significantly altered in EcRB1 RNAi larvae (Fig 6C right).

Larvae in which the expression of *ultraspiracle* (*usp*), a co-receptor for EcR, was suppressed by RNAi in the class IV neurons, were significantly hyposensitive to noxious thermal stimuli (Fig 2F), but not to noxious mechanical stimuli (Fig 3F). The class IV ddaC cells of USP hypomorphs did not show any significant changes in dendritic branch number or length (Fig 6D).

## Discussion

Steroid modulation of pain sensitivity is well known in mammals [8], but a detailed understanding of the mechanisms awaits a systematic investigation. The *Drosophila* system offers a simplified model in which to explore steroid effects using genetic approaches.

Ecdysone receptor activity is known to be integral to neuronal development [22]. For example, Zwart and coworkers [9] demonstrated that receptor isoform EcRB2 controls the expansion of the dendritic arbors of larval motor neurons. In a cell-specific fashion, EcRB2 maintains the appropriate activity levels of the motor neurons, ensuring functional integrity of the circuitry as the sizes of the various components increase. Similarly, the normal dendritic structure of one type of sensory neuron, the class I multidendritic neuron, has also been shown to require EcR [13].

Using a cell-specific approach we demonstrate that *EcR* expression by another type of neuron, the nociceptive class IV sensory neuron, is required for normal dendritic architecture (Figs 5 and 6), and is also required for normal function of these neurons (Figs 2 and 3). The observed hyposensitivity to noxious thermal stimulation that results from *EcR* suppression in these cells suggests that EcR is responsible for regulating their sensitivity. Since several *EcR* genotypes, including multiple distinct RNAi genotypes and one temperature-sensitive allele, all display similar hyposensitivity phenotypes, these observations are not likely to be the result of off-target RNAi effects. Results of experiments in which EcR’s co-receptor ultraspiracle (USP) was reduced roughly agreed with the behavioral results of EcR reduction (Figs 2F and 3F). However the effects of *usp* knockdown were less robust, perhaps due to possibly incomplete penetrance by the RNAi allele used.

EcRA and EcRB1 were both detected in the nuclei of third instar class IV multidendritic neuron via immunohistochemical techniques (Fig 1). We detected significant reductions in EcRA and EcRB1 staining in RNAi larvae (p<0.0001 and p < 0.021, respectively, see S1 Dataset).

The class IV multidendritic neurons are required not only for sensitivity to noxious stimuli, but also for normal locomotion [17]. To confirm that larvae of experimental genotypes are capable of locomotion, and therefore that their hyposensitivity phenotype is not actually due to paralysis, their spontaneous motor activity was measured. Using an automated optical recording method [20], locomotion of larvae bearing RNAi alleles of *EcR* was observed to be similar to or greater than that of controls (Fig 4B–4E). In animals with EcR activity reduced globally using the temperature sensitive allele *EcR* ^*A483T*^ (Fig 4A), spontaneous activity, while robust, was lower than that of controls. This observation may reflect the consequences of reducing EcR activity not only in class IV multidendritic neurons, but also in other behaviorally relevant cells such as motor neurons and myocytes, the net result being reduced activity. The possibility nonetheless exists that in this group, the observed hyposensitivity to noxious stimulation could reflect an obscuring hypoactivity instead. In contrast, animals bearing an RNAi allele that targets all EcR isoforms specifically in nociceptor neurons demonstrate a significant increase in activity (Fig 4B). This hyperactivity is unlikely to confound the conclusion that these mutants are hyposensitive to noxious stimulation (Figs 2C and 3C).

The results, summarized in Table 1, suggest two mechanisms by which EcR may regulate sensitivity of these cells to noxious stimulation. EcRA deficiency leads to a reduction in sensitivity and class IV ddaC dendrite length, but only a nominal reduction in branch number, not rising to the level of statistical significance (Fig 6B). On the other hand, deficiency in EcRB1 leads to a significant reduction of both ddaC dendrite branching and length (Fig 6C) with nominal but not statistically significant changes in sensitivity to noxious stimuli measured behaviorally (Figs 2E and 3E). When both EcRA and EcRB1 (and presumably EcRB2) are reduced with RNAi targeting all EcR isoforms, both sensitivity (Figs 2B, 2C, 3B and 3C) and ddaC dendrite morphology are robustly reduced (Figs 5 and 6A). It may be that EcRA and EcRB1 have overlapping roles in controlling the sensitivity of these class IV nociceptor neurons, but each isoform is biased toward control of distinct mechanisms of sensitivity. EcRA seems to be somewhat less critical for modulating dendrite architecture in that suppression of EcRA reduces overall length but not branching (Fig 6B), while at the same time being necessary for normal sensitivity (Figs 2D and 3D). It is possible that EcRA promotes sensitivity of these neurons by some intrinsic mechanism that is separate from the machinery that controls dendritic innervation, such as enhancing the expression of nociceptive TRP channels [23]. In contrast, EcRB1 appears to be indispensible for normal dendrite architecture of the class IV ddaC cells (Fig 6C), such that EcRB1 suppression leads to a reduced dendritic arbor, without strongly compromising sensitivity to noxious stimuli (Figs 2E and 3E). Zwart and coworkers demonstrated a similar inability of a single EcR isoform to control multiple aspects of cell physiology by observing that while the growth of the larval motor neuron’s dendritic arbor is under the control of EcRB2, the electrophysiology of the cell is apparently not.

**Table 1:** Summary of consequences of nociceptor-specific RNAi suppression of EcR isoforms and USP.

	Thermal sensitivity	Mechanical sensitivity	Dendritic morphology	Spontaneous locomotion
**EcR RNAi**	Reduced	Reduced	Reduced length and branching	Increased
**EcRA RNAi**	Reduced	Reduced	Reduced in length only	Not different
**EcRB1 RNAi**	Not different	Not different	Reduced length and branching	Not different
**USP RNAi**	Reduced	Not different	Not different	Not different

It would be interesting to know the mechanism by which EcR, particularly EcRB1, deficiency leads to dendrite hypomorphy in the class IV neurons including ddaC. One possibility is that EcR signaling is required for the initial development of a normal dendritic arbor in these cells, and that by impairing EcR activity, the arbor is caused to develop abnormally. On the other hand, perhaps the dendrite architecture is dynamic at this point in larval development, and EcR is required for maintenance of a normal balance of dendrite extension and removal. The mechanical hyposensitivity observed in *elav-Geneswitch/UAS-EcR-RNAi* larvae whose *EcR* was suppressed after presumably normal development to the third instar (Fig 3B) provides some support for this latter hypothesis. In either case, EcR seems to affect only the scale of the arbor, since complexity, as represented by the ratio of branch number to length, remains unaltered in the genotypes analyzed (Fig 6 right).

In addition to its role in controlling dendritic growth during larval life, as discussed above, ecdysone signaling is known to be required for the complete pruning of the dendrites of class IV ddaC neurons in early metamorphosis [24]. A new arbor develops to serve the new adult tissues. To achieve this dramatic change in dendritic morphology, EcR isoform EcRB1 upregulates *Sox14* which, acting through *mical* leads to the removal of dendrites at this point. The chromatin effector CREB binding protein is also required. In contrast to studies establishing the role of EcRB1 in triggering dendrite removal during metamorphosis [14], our results suggest that prior to metamorphosis, EcRB1 promotes full dendritic arborization, in that impairment of its expression in ddaC neurons causes dendritic reduction (Fig 6C). In other words, after bringing about full development of the arbors of sensory neurons like ddaC during larval life, a metamorphic switch is activated to reverse the pro-dendritic effects of ecdysone signaling through EcRB1, resulting in dendritic removal. Perhaps this switch is related to the decline in the level of juvenile hormone that occurs as the larva approaches metamorphosis [25].

Since it appears that EcRA and EcRB1 might act through different processes to regulate the observed hyposensitivity to noxious stimuli, we are now interested in understanding the specifics of each process. EcRB1’s effect on dendrite architecture may be in its initial development, or its maintenance, and may be potentially independent of the pruning associated with metamorphosis. In contrast, EcRA may regulate cellular sensitivity systems that may be in addition to or partially overlapping with those regulating dendrite architecture. We are curious about the specifics of the pathways by which steroid hormones may modulate sensitivity to noxious stimuli, which may lead to novel analgesic treatments for pain.

## Materials and Methods

### Fly Stocks and Genetics

Fly stocks were maintained at 25°C unless otherwise indicated, in a 12h light: 12h dark cycle throughout the duration of the experiments. All genotypes were reared on standard cornmeal-yeast-sucrose diet. We used the GAL4/UAS system to drive the expression of UAS transgenes in the larvae both pan-neuronally and specifically in class IV nociceptive sensory neurons. A *ppk1*.*9-Gal4* driver line also bearing *ppk-eGFP* was generously provided by Dr. Michael Galko. *UAS-EcR* ^*IR*^(BDSC#9327), *UAS-USP* ^*IR*^(BDSC#9328), *UAS-EcRA* ^*IR*^ (BDSC#9329) and *UAS-EcRB1* ^*IR*^ (BDSC#27258) were used to investigate the effects of reduced receptor expression on nociceptive avoidance response. *UAS-EcR* ^*IR*^ was in some experiments driven by a neuron-specific *elav-Geneswitch* driver (generously provided by Dr. Yick-Bun Chan), which requires larvae to be reared in the presence of 12 μg/ml mifepristone to activate the chimeric GAL4 Geneswitch protein [19]. Each GAL4/UAS experimental genotype was compared with two parental controls, one being the progeny of the *Gal4* driver was crossed with a line representing the genetic background of the UAS line, either *w* ^*1118*^ or *y* ^*1*^ *v* ^*1*^. The other parental control consisted of the progeny of the UAS responder line crossed with *w* ^*1118*^. In some experiments, EcR activity was reduced globally using a null allele combined with a temperature-sensitive allele (*EcR* ^*M554fs*^ / *EcR* ^*A483T*^; BDSC #4894 and #5799 respectively [18]. Larvae of this genotype were raised at the permissive temperature (22^°^C) and treated at the restrictive temperature (29^°^C) for 24 hours before assaying. In all experiments, large foraging third instar larvae were selected for analysis.

### Larval Nociceptive Behavior Assays

Noxious stimuli were administered using a mechanical probe [26] or a thermal probe [27] (ProDev Engineering, Missouri City, TX) by an operator blind to genotype and/or treatment. The mechanical probe consisted of a lever supporting a nitinol filament calibrated by depressing the filament tip onto an electronic balance until the filament bent. The length of the filament was then adjusted to generate the desired force of 45mN. The thermal probe was set to deliver a temperature of 45°C, previously used to measure baseline nociceptive responses [27]. Both noxious modalities were administered along the dorsal midline between abdominal segments A2 and A5. Withdrawal behavior was defined as at least one complete 360° roll in response to the stimulus. For thermal stimuli, response latency was recorded, and responses were categorized as previously described [27]: 0–6 seconds as fast, 6–20 seconds as slow and more than 20 seconds as no response. For mechanical stimulation, responses above threshold are instantaneous, therefore avoidance was scored as either response or no response. All data are available in the supplementary file S1 Dataset.

### Morphometry

Class IV multidendritic neurons were visualized using a live-imaging technique [28]. Larvae were anesthetized using a halocarbon ether mix (2:1) and imaged on a Leica SP5 Scanning Confocal Microscope. Images were obtained using a 20x dry lens. Z-stacks were compiled with a step size of 1.5 μm. Image resolution was set to 1024x1024. All settings including laser power, gain and pinhole width were maintained across all groups. The n-value was 15 or more for each group. Maximum projections were obtained from the z-stacks and analyzed in Fiji (http://fiji.sc/Fiji). Skeletons were constructed and total dendritic length and total number of branches were calculated. Statistical analyses were performed using Instat (Graphpad Software). Data were analyzed using Students t-test, and are available in the supplementary file S1 Dataset.

### Immunohistochemistry

To visualize EcR protein expression, animals were processed using a larval fillet dissection technique [29]. Individual third instar larvae carrying a *ppk-eGFP* promoter-reporter fusion to identify class IV neurons were washed in deionized water and placed onto a Sylgard-lined petri dish containing PBST. Larvae were then pinned at the head and tail regions using stainless steel minutien pins (Fine Science Tools) cut to 4-5mm. Larvae were slit longitudinally at the ventral midline with iris scissors and all visceral tissues were removed including major tracheae. Pins were then used to open up larvae in the shape of a square fillet, cuticle side facing down. Dissection solution was changed to fresh 4% paraformaldehyde in PBS, and fixed for 20 minutes, followed by three 10-minute washes in PBST. Fillets were blocked in 5% normal goat serum for 4 hours, incubated with anti-EcR monoclonal antibodies (15G1a (EcRA), AD4.4 (EcRB1) both at 1:50; Developmental Studies Hybridoma Bank) overnight at 4°C and detected with fluorescent goat anti-mouse secondary antibodies. Samples were imaged using a Leica SP5 Scanning Confocal Microscope. Images were taken with 40x oil-immersion lens. Resolution was set to 1024x1024. All settings including laser power, gain and pinhole width were maintained across all groups. Z-stacks were produced to encompass the cell body.

### Activity Measurements

Spontaneous locomotor activity of the various genotypes was measured optically with a previously described larval activity assay, using the *Drosophila* activity monitor (DAM; Trikinetics) [20]. Larvae were collected using a mesh filter as above, and were individually placed into humidified glass tubes sealed with agar plugs. Tubes were then placed into the DAM (in a 20°C incubator) and activity data were collected for twenty minutes. Data and analysis are available in the supplementary file S1 Dataset.

### Statistical Analysis

For nociception assays, we used Fisher’s exact tests to compare categorical response: no response within 20s cutoff, slow response (between 6 seconds and 20 seconds), or fast response (under 6 seconds). A Student’s t-test was used to determine differences in morphological parameters of dendritic arbors.

## Supporting Information

S1 Dataset Complete dataset used to reach the conclusions drawn in the article. This spreadsheet is arranged such that each tab contains the relevant data supporting a particular figure or part of a figure.
(XLSX)
